# Two-Qubit Entanglement Generation through Non-Hermitian Hamiltonians Induced by Repeated Measurements on an Ancilla

**DOI:** 10.3390/e22101184

**Published:** 2020-10-20

**Authors:** Roberto Grimaudo, Antonino Messina, Alessandro Sergi, Nikolay V. Vitanov, Sergey N. Filippov

**Affiliations:** 1 Dipartimento di Fisica e Chimica dell’Università di Palermo, Via Archirafi 36, I-90123 Palermo, Italy; 2 Dipartimento di Matematica ed Informatica dell’Università di Palermo, Via Archirafi 34, I-90123 Palermo, Italy; antonino.messina@unipa.it; 3 Istituto Nazionale di Fisica Nucleare, Sez. di Catania, 95123 Catania, Italy; alessandro.sergi@unime.it; 4 Dipartimento di Scienze Matematiche e Informatiche, Scienze Fisiche e Scienze della Terra, Università degli Studi di Messina, viale F. Stagno d’Alcontres 31, 98166 Messina, Italy; 5 Institute of Systems Science, Durban University of Technology, P. O. Box 1334, Durban 4000, South Africa; 6 Department of Physics, St. Kliment Ohridski University of Sofia, 5 James Bourchier Boulevard, 1164 Sofia, Bulgaria; vitanov@phys.uni-sofia.bg; 7 Steklov Mathematical Institute of Russian Academy of Sciences, Gubkina St. 8, Moscow 119991, Russia; sergey.filippov@phystech.edu

**Keywords:** entanglement generation, zeno effect, non-Hermitian Hamiltonians

## Abstract

In contrast to classical systems, actual implementation of non-Hermitian Hamiltonian dynamics for quantum systems is a challenge because the processes of energy gain and dissipation are based on the underlying Hermitian system–environment dynamics, which are trace preserving. Recently, a scheme for engineering non-Hermitian Hamiltonians as a result of repetitive measurements on an ancillary qubit has been proposed. The induced conditional dynamics of the main system is described by the effective non-Hermitian Hamiltonian arising from the procedure. In this paper, we demonstrate the effectiveness of such a protocol by applying it to physically relevant multi-spin models, showing that the effective non-Hermitian Hamiltonian drives the system to a maximally entangled stationary state. In addition, we report a new recipe to construct a physical scenario where the quantum dynamics of a physical system represented by a given non-Hermitian Hamiltonian model may be simulated. The physical implications and the broad scope potential applications of such a scheme are highlighted.

## 1. Introduction

Historically, Gamow [1] was the first to adopt a non-Hermitian Hamiltonian in order to study the radiative decay of nuclei. There are also a number of other instances where non-Hermitian Hamiltonians are useful [2]. For example, this happens when one wants to study the parity–time (PT) symmetry properties of the Hamiltonian [3]. Another theory formulated in terms of non-Hermitian Hamiltonians is obtained through the introduction of complex scaling transformations [2]. Effective non-Hermitian Hamiltonians are also obtained when, from a space comprising discrete and continuous states, the continuous states are projected out [4,5,6]. The Fock and Krylov theorem [7] states that the necessary and sufficient condition for the presence of true decaying states is that there must be a continuous part of the spectrum. Hence, the projection operator formalism [4,5,6] and non-Hermitian Hamiltonians provides an effective way to describe decaying states. Non-Hermitian Hamiltonians can also be postulated on the basis of physical considerations [8,9,10,11,12], in order to describe gain or loss of probability. Dynamics in terms of non-Hermitian Hamiltonians have been investigated for quantum [13] and quantum-classical systems [14], adopting phase space representation quantum mechanics. The dynamics of non-Hermitian quantum mechanical systems can be studied either in terms of a linear equation for a non-normalized density matrix [8,15] or in terms of a non-linear equation for a normalized density matrix [8,15]. Upon combining the linear evolution for the non-normalized density matrix and the non-linear equation of motion for the normalized density matrix, different forms of correlations functions [16] and entropies [17,18] have been defined.

Despite such developments in the theoretical realm, so far the observation of non-Hermitian dynamics in experimental situations has been somewhat limited to classical dissipative systems whose theoretical description was mapped onto that provided by quantum-like non-Hermitian (and often PT-symmetric) Hamiltonians. Examples of such systems are given by optical lattices [19,20], optical radiation interacting with atomic systems [21,22,23], electronic circuits [24,25,26], microwave billiards [27] simple mechanical systems [28], and acoustical systems [29,30,31]. In all these cases, the non-Hermitian dynamics of classical systems are well understood and experimentally realized by means of asymmetric attenuation and amplification. However, the experimental realization of true non-Hermitian quantum systems (i.e., which do not arise from an isomorphism between classical dissipative dynamics and non-Hermitian quantum mechanics) is difficult since quantum systems naturally obey the laws of Hermitian quantum mechanics. For example, both the attenuation and amplification of signals are described by physical quantum channels (completely positive and trace preserving maps), with Hermitian Hamiltonian dynamics involving the system and its environment [32,33,34].

In order to demonstrate the occurrence of non-Hermitian Hamiltonians, some theoretical methods have been proposed. Among these, we highlight those based on the universal concept of dilation mapping [35] of a non-Hermitian Hamiltonian into a Hermitian Hamiltonian living in a higher dimensional Hilbert space [36,37,38,39,40]. Interestingly, the dilation mapping is, broadly speaking, the inverse of the projection formalism [4,5,6], according to which one projects a Hermitian Hamiltonian into a non-Hermitian Hamiltonian, defined in a lower dimensional Hilbert space. Although all the known schemes exploit the general concept of dilation/inverse-projection formalism in order to propose experimental schemes for building non-Hermitian Hamiltonians, the actual implementation of these schemes is tailored in some way to a chosen, specific system. For instance, the authors of [40] use a time-dependent Hermitian Hamiltonian in a higher dimensional Hilbert space of two qubits in order to simulate a non-Hermitian Hamiltonian for a single qubit.

Quite recently, an experimental scheme implementing the quantum dynamics of a finite-dimensional system *S* generated by a non-Hermitian Hamiltonian operator, has been reported [41]. The basic idea is to couple *S* with a quantum ancilla subsystem *A* and to follow the time evolution of *S* conditioned by a Zeno measurement protocol applied on the ancilla finite-dimensional subsystem only. In accordance with the previosly quoted [41], the reduced density matrix of *S*, conditioned by the progression of collapses induced in this way on the state of the combined system S+A, evolves under the action of an effective non-Hermitian Hamiltonian which may be explicitly constructed in the so called stroboscopic limit. We observe that the proposal of [41] differs from that of Feshbach since the latter is not a conditional one and, moreover, the ancilla subsystem can hardly be considered dynamically equivalent to an environment with infinite degrees of freedom, as requested by Feshbach in in his projection method. In addition, the scheme proposed in [41] can be easily experimentally implemented and, form a theoretical point of view, leads to a solvable quantum dynamical problem.

Considering the idea of [41], it is interesting to note the following. Continuous measurements on the ancilla generate infinitesimal lifetimes for its states. Hence, the time–energy uncertainty principle makes sure that the energy of the ancilla under continuous measurements cannot be sharply peaked. It follows that the ancilla under continuous measurements effectively acts as a continuum of states with which the system *S* interacts. A similar reasoning is found in [42]. According to the theorem of Fock and Krylov [7], once the system is in contact with a continuum of states, provided by the ancilla under continuous measurements, it evolves, experiencing the decay of its states. The limited lifetime of decaying states [43] and the representation of the width of the energy levels, by means of an imaginary component of the system’s eigenvalues, naturally lead to a non-Hermitian Hamiltonian.

The first goal of this work is to prove theoretically that entanglement in a system of two interacting qubits can be generated by means of stroboscopic measurements on a third qubit, coupled to the first two, for which it constitutes the ancilla subsystem *A* (requested by [41]). Continuous measurements on the ancilla, that is, a Zeno measurement protocol, produce an effective non-Hermitian Hamiltonian determining the time evolution of the reduced and conditioned density matrix of the two-qubit system *S*. Two main results must be emphasized: (1) the possibility of generating maximally entangled states of the two qubits thanks to the repeated measurements on the ancilla; (2) the possibility of getting information about the (an)isotropy level of the pairwise interactions between the three qubits, by studying the effective dynamics of the two-qubit system.

The experimental protocol reported in [41] (hereafter referred to as direct) realizes a dynamical constraint under which the system *S* is effectively driven in its Hilbert space as if it were subjected to a non-Hermitian Hamiltonian model. In this paper, we successfully face the following inverse problem: given a non-Hermitian Hamiltonian model at will, describe the quantum dynamics of a physical system *S*, to find a Hermitian model reproducing the assigned non-Hermitian Hamiltonian of the direct procedure presented in [41]. In principle, solving this inverse problem means associating to an arbitrary non-Hermitian model a physical scenario where its quantum dynamics can be experimentally simulated.

This manuscript is organized in the following way. In Section 2, the formalism describing the time evolution of the reduced density matrix when the Hamiltonian of the relevant system is non-Hermitian is outlined. In Section 3, we discuss at length the general direct scheme for experimentally realizing an (a priori unknown) non-Hermitian Hamiltonian. In Section 4, the recipe for solving the inverse problem is reported. The application of the direct protocol to a (two+one)-qubit system (S+A) model and a detailed study of the effective non-Hermitian dynamics of the resulting two-qubit system are developed in Section 5, where remarkable physical effects, suitable for experimental and technological applications, are brought to light. Finally, conclusive remarks and comments are reported in the last section.

## 2. Density Matrices and Effective Non-Hermitian Hamiltonians

Let us assume that the dynamics of a quantum system *S*, living in a discrete Hilbert space, are described by a non-Hermitian Hamiltonian, $H_{\mathrm{eff}}\neq H_{\mathrm{eff}}^{†}$. If *S* is appropriately coupled to an environment living in a continuous Hillbert space, the projection operator formalism [4,5,6] allows one to derive such a non-Hermitian Hamiltonian describing the system [44]. Then, in terms of the non-Hermitian Hamiltonian [45] $H_{\mathrm{eff}}$, the Schrödinger equation reads

(1) $$\frac{d}{dt}|\;\Psi \left(t\right)\;\rangle =-iH_{\mathrm{eff}}|\;\Psi \left(t\right)\;\rangle .$$

In Equation (1) and in the following, we assume units of measurement such that ℏ=1. In accordance with the theorem of Fock and Krylov [7], stating that the necessary and sufficient condition for the existence of true decaying states for *S* is the interaction with a continuum of states, Equation (1) describes the decay of the system’s states. As a matter of fact, for each solution |Ψ(t)〉, the solution of a Cauchy problem for Equation (1) can also be written in terms of a non-unitary propagator $U\left(t\right)\neq U^{†}\left(t\right)$ [42,45]:

(2) |Ψ(t)〉=U(t)|Ψ(0)〉,

which clearly shows that the probability for the system is not conserved. Upon defining a non-normalized density matrix as ρ=|Ψ(t)〉〈Ψ(t)|, one can easily derive its equation of motion:

(3) $$\frac{d}{dt}\rho \left(t\right)=-\frac{i}{\hbar }\left(H_{\mathrm{eff}}\rho \left(t\right)-\rho \left(t\right)H_{\mathrm{eff}}^{†}\right)\;,$$

whose solution can be written as $U\left(t\right)\rho \left(0\right)U^{†}\left(t\right)$, as usual. When $H_{\mathrm{eff}}$ is time-independent (as we will assume), the non-unitary propagator introduced in Equation (2) can be written as $U\left(t\right)=\exp\left(-iH_{\mathrm{eff}}t\right)$.

The non-Hermitian Hamiltonian $H_{\mathrm{eff}}$ can always be defined in terms of the sum of a Hermitian Hamiltonian, $H_{0}=\left(H_{\mathrm{eff}}+H_{\mathrm{eff}}^{†}\right)/2$ and an anti-Hermitian operator, $i\Gamma =-(H_{\mathrm{eff}}-H_{\mathrm{eff}}^{†})/2$. The Hermitian operator Γ is called the decay operator. Combining Equation (1) with its adjoint [46], one obtains the equation of motion [8] for the density matrix introduced right above Equation (3)

(4) $$\begin{array}{c} \frac{d}{dt}\rho \left(t\right)=-\frac{i}{\hbar }\left[H_{0},\rho \left(t\right)\right]-\frac{1}{\hbar }\left\{\Gamma ,\rho (t)\right\}\;, \end{array}$$

where [·,·] is the commutator and {·,·} is the anti-commutator. Equations (3) and (4) reduce to the standard ones when Γ=0 (which means that $H_{\mathrm{eff}}=H_{0}$ is Hermitian). Equations (3) and (4) keep their validity even if the system is initially not in a pure state |Ψ(0)〉 but in a mixture of states $|\;\Psi^{\left(i\right)}\left(0\right)\;\rangle$.

Equations (3) and (4) do not conserve the trace of the non-normalized density matrix ρ(t). Upon taking the trace of Equation (4), one gets

(5) $$\begin{array}{c} \frac{d}{dt}\mathrm{Tr}\left[\rho (t)\right]=-\frac{2}{\hbar }\mathrm{Tr}\left[\Gamma \rho (t)\right]\;. \end{array}$$

Equation (5) shows explicitly that the probability of the system is not conserved. Upon normalizing ρ(t) at every *t* with its time-dependent trace, one can define a normalized density matrix ϱ(t) given by

(6) $$\begin{array}{c} \varrho \left(t\right)=\frac{\rho (t)}{\mathrm{Tr}\left[\rho (t)\right]}=\frac{e^{-iH_{\mathrm{eff}}t}\rho \left(0\right)e^{iH_{\mathrm{eff}}^{†}t}}{\mathrm{Tr}\left[e^{-iH_{\mathrm{eff}}t}\rho \left(0\right)e^{iH_{\mathrm{eff}}^{†}t}\right]}=\frac{U\left(t\right)\rho \left(0\right)U^{†}\left(t\right)}{\mathrm{Tr}\left[U\left(t\right)\rho \left(0\right)U^{†}\left(t\right)\right]}\;. \end{array}$$

The equation of motion obeyed by ϱ(t) is [8,10,15,16,17]

(7) $$\frac{d}{dt}\varrho \left(t\right)=-\frac{i}{\hbar }\left[H_{0},\varrho \left(t\right)\right]-\frac{1}{\hbar }\left\{\Gamma ,\varrho (t)\right\}+\frac{2}{\hbar }\mathrm{Tr}\left[\Gamma \varrho (t)\right]\varrho \left(t\right)\;.$$

Equation (7) is a non-linear equation that, by construction, preserves the trace of ϱ(t). Averages of dynamical variables, which are represented by operators denoted here with χ, are calculated in the the standard way

(8) $$\langle \chi \left(t\right)\rangle \equiv \mathrm{Tr}\left[\varrho (t)\chi \right]\;.$$

When the operator χ is Hermitian, the average in Equation (8) is real. Equation (7) reduces to the standard linear equation of Hermitian quantum mechanics when Γ=0.

Equations (1)–(8) imply that, notwithstanding the non-Hermitian Hamiltonian $H_{\mathrm{eff}}$, non-linear Hermitian quantum mechanics can be defined. In the next Section, it is shown that the structure of the nonlinear equation in (7) turns out to be useful in a different context too, where the system is coupled to a continuously measured ancilla.

## 3. Non-Hermitian Hamiltonians Due to Repeated Measurements

**Figure 1:**
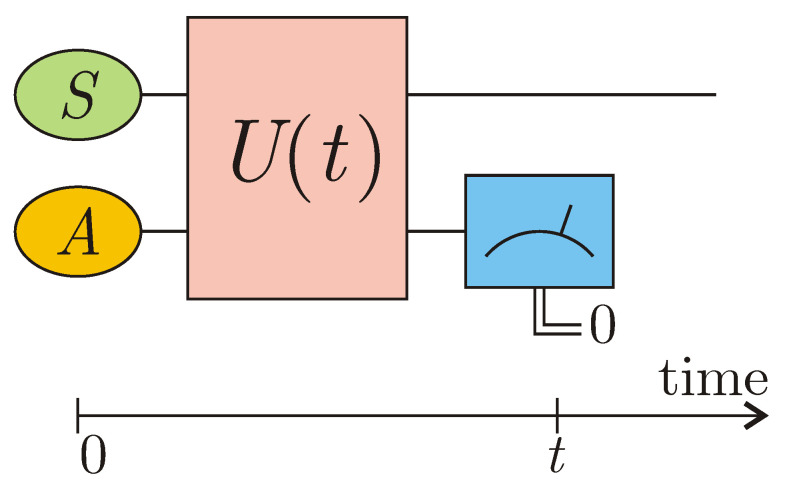
Conditional implementation of non-unitary dynamics for system *S* via projective measurement on ancilla *A*.

When one studies a bipartite system undergoing unitary quantum dynamics, there is the possibility to physically and conditionally implement a non-unitary dynamics for one of its subsystems, with the resulting effective Hamiltonian being non-Hermitian [40,41,47]. The idea is to let a quantum system *S* interact with an ancillary quantum system *A* for some time *t* and then perform a projective measurement on the ancilla; see Figure 1. The Hamiltonian operator *H*, describing such a coupling, is supposed to be time-independent and experimentally implementable. Provided the ancilla is originally prepared in the pure non-degenerate state $|\;0_{A}\;\rangle$ and measured in the orthonormal basis $\left\{|\;0_{A}\;\rangle ,\ldots \right\}$, with the result being 0, the reduced density operator of the system *S* at the time *t* collapses into the following un-normalized conditional density matrix:

(9) $$\begin{array}{cc} \rho_{S}\left(0\right)⟶\rho_{S}^{c}\left(t\right) & =\langle \;0_{A}\;|\;\;U\left(t\right)\;\;\left[\rho_{S}\left(0\right)⊗|\;0_{A}\;\rangle \langle \;0_{A}\;|\right]\;\;U^{†}\left(t\right)\;\;|\;0_{A}\;\rangle \\ \; & =[\langle \;0_{A}\;|\;\;U\left(t\right)|\;0_{A}\;\rangle ]\;\;\langle \;0_{A}\;|\left[\rho_{S}\left(0\right)⊗|\;0_{A}\;\rangle \langle \;0_{A}\;|\right]|\;0_{A}\;\rangle \;\;[\langle \;0_{A}\;|U^{†}\left(t\right)\;\;|\;0_{A}\;\rangle ]\\ \; & \equiv K\left(t\right)\rho_{S}\left(0\right)K^{†}\left(t\right), \end{array}$$

where, in the first equality, we introduced the identity operator $I_{A}=\sum_{j}|\;j_{A}\;\rangle \langle \;j_{A}\;|$, while, in the last passage, we defined in the Hilbert space of the system *S*, the operator $K\left(t\right)\equiv \langle \;0_{A}\;|\;U\left(t\right)\;|\;0_{A}\;\rangle$. $\rho_{S}\left(0\right)$ represents the initial reduced density matrix of the subsystem *S*, while $\rho_{S}^{c}\left(t\right)$ stands for the non-normalized reduced conditional density matrix stemming from the measurement act performed on the ancilla qubit at t>0. U(t) is the unitary evolution operator for the whole system S+A, governed by *H*, while K(t), as a submatrix of a unitary matrix, is a non-unitary evolution operator for the system *S*. Thus, $\rho_{S}^{c}\left(t\right)$ is not a density matrix since its evolution does not preserve its trace. The success probability to observe the outcome 0 while measuring the ancilla qubit at the time *t* is

(10) $$p_{\rho_{S}\left(0\right)}\left(t\right)=\langle \;0_{A}\;|\rho_{A}\left(t\right)|\;0_{A}\;\rangle =\langle \;0_{A}\;|\;\;{\mathrm{tr}}_{S}\left\{U\left(t\right)\;\;\left[\rho_{S}\left(0\right)⊗|\;0_{A}\;\rangle \langle \;0_{A}\;|\right]\;\;U^{†}\left(t\right)\right\}\;\;|\;0_{A}\;\rangle ={\mathrm{tr}}_{S}\rho_{S}^{c}\left(t\right)\equiv \mathrm{tr}\rho_{S}^{c}\left(t\right),$$

and depends on the initial system state $\rho_{S}\left(0\right)$. The trace of the subnormalized operator $\rho_{S}^{c}\left(t\right)$ determines how often the desired event takes place. The properly normalized density operator reads $\varrho_{S}^{c}\left(t\right)=\rho_{S}^{c}\left(t\right)/\mathrm{tr}\rho_{S}^{c}\left(t\right)=\rho_{S}^{c}\left(t\right)/p_{0}\left(t\right)$.

It is worthwhile pointing out that the authors of [40] simulate the qubit evolution with a time-independent non-Hermitian Hamiltonian $H_{\mathrm{eff}}$ by controlling the actual evolution operator U(t) for the system and ancilla. In this case, the Hamiltonian for the system and ancilla is time-dependent because U(t) is not a semigroup that requires sophisticated driving. Repeated measurements on ancilla help overcome this drawback in the stroboscopic limit [41], when the dynamics of ancilla are effectively frozen.

**Figure 2:**
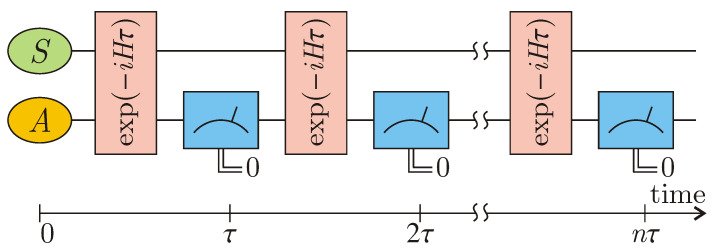
Repeated measurements on ancilla *A* result in non-Hermitian Hamiltonian dynamics for system *S*.

Suppose the ancilla is initially in the non-degenerate state $|\;0_{A}\;\rangle$ and is repeatedly measured after equal time intervals τ on the basis of $\left\{|\;0_{A}\;\rangle ,\ldots \right\}$, see Figure 2. By considering the physical meaning of Equation (9), $\rho_{S}^{c}\left(\tau \right)$ determines the new initial state of *S* which, when tensorially multiplied by $|\;0_{A}\;\rangle \langle \;0_{A}\;|$, gives the new (non-normalized) initial condition for the total system S+A after the measurement act at the time instant τ. Provided that *n* sequential measurements give the outcome 0, the system state, in view of Equation (9), collapses into

(11) $$\rho_{S}\left(0\right)⟶\rho_{S}^{c}\left(n\tau \right)=K^{n}\left(\tau \right)\rho_{S}\left(0\right){\left(K^{n}\left(\tau \right)\right)}^{†}.$$

The probability p(nτ) for observing *n* sequential outcomes 0 while measuring the ancilla qubit *n* times is

(12) $$\begin{array}{ccc} p(n\tau ) & = & p_{\rho_{S}\left(0\right)}\left(\tau \right)\times p_{\varrho_{S}^{c}\left(\tau \right)}\left(\tau \right)\times \ldots \times p_{\varrho_{S}^{c}\left(\left(n-1\right)\tau \right)}\left(\tau \right)\\ & = & \mathrm{tr}\left[K\left(\tau \right)\rho_{S}\left(0\right)K^{†}\left(\tau \right)\right]\times \mathrm{tr}\left[K\left(\tau \right)\varrho_{S}^{c}\left(\tau \right)K^{†}\left(\tau \right)\right]\times \ldots \times \mathrm{tr}\left[K\left(\tau \right)\varrho_{S}^{c}\left(\left(n-1\right)\tau \right)K^{†}\left(\tau \right)\right]\\ & = & \mathrm{tr}\left[K\left(\tau \right)\rho_{S}\left(0\right)K^{†}\left(\tau \right)\right]\times \frac{\mathrm{tr}[K\left(\tau \right)\rho_{S}^{c}\left(\tau \right)K^{†}\left(\tau \right)]}{\mathrm{tr}[K\left(\tau \right)\rho_{S}\left(0\right)K^{†}\left(\tau \right)]}\times \ldots \times \frac{\mathrm{tr}[K\left(\tau \right)\rho_{S}^{c}\left(\left(n-1\right)\tau \right)K^{†}\left(\tau \right)]}{\mathrm{tr}[K\left(\tau \right)\rho_{S}^{c}\left(\left(n-2\right)\tau \right)K^{†}\left(\tau \right)]}\\ & = & \mathrm{tr}\left[K\left(\tau \right)\rho_{S}^{c}\left(\left(n-1\right)\tau \right)K^{†}\left(\tau \right)\right]=\mathrm{tr}\left[K^{n}\left(\tau \right)\rho_{S}\left(0\right){\left(K^{n}\left(\tau \right)\right)}^{†}\right]=\mathrm{tr}\rho_{S}^{c}\left(n\tau \right), \end{array}$$

which means that p(nτ) is merely the trace of the unnormalized operator $\rho_{S}^{c}\left(n\tau \right)$ and monotonously diminishes.

Since the Hamiltonian of the combined closed system S+A is time independent, the operator $K\left(\tau \right)=\langle \;0_{A}\;|\exp\left(-iH\tau \right)|\;0_{A}\;\rangle$ is time-independent as well. The Taylor expansion with respect to τ yields

(13) $$\begin{array}{ccc} K(\tau ) & = & \langle \;0_{A}\;|\left(I_{S+A}-i\tau H-\frac{\tau^{2}}{2}H^{2}+o_{S+A}\left(\tau^{2}\right)\right)|\;0_{A}\;\rangle \\ & = & I_{S}-i\tau \langle \;0_{A}\;|H|\;0_{A}\;\rangle -\frac{\tau^{2}}{2}\langle \;0_{A}\;|H^{2}|\;0_{A}\;\rangle +o_{S}\left(\tau^{2}\right)\\ & = & \exp\left(-i\tau H_{0}^{S}-\frac{\tau^{2}}{2}\Gamma_{S}+o_{S}\left(\tau^{2}\right)\right), \end{array}$$

where $o(\tau^{2})$ denotes an operator acting on the corresponding Hilbert space (S+A or *S*) such that its norm $∥o(\tau^{2})∥$ satisfies $\lim_{\tau \to 0}∥o\left(\tau^{2}\right)∥/\tau^{2}=0$,

(14) $$H_{0}^{S}={\left(H_{0}^{S}\right)}^{†}=\langle \;0_{A}\;|H|\;0_{A}\;\rangle \;\mathrm{and}\;\Gamma_{S}=\Gamma_{S}^{†}=\langle \;0_{A}\;|H^{2}|\;0_{A}\;\rangle -{\left(H_{0}^{S}\right)}^{2}=\langle \;0_{A}\;|H|\;1_{A}\;\rangle \;\langle \;1_{A}\;|H|\;0_{A}\;\rangle \geq 0.$$

If the measurement repetition rate $\frac{1}{\tau }$ is much greater than the maximum Bohr frequency of *H*, then one can neglect the term $o_{S}\left(\tau^{2}\right)$ in Equation (13). This means that the system evolution in between the measurement acts is infinitesimal, so that the stroboscopic time t=nτ is quasi-continuous in full analogy with the quantum collision models [48,49,50,51]. As a result, we obtain

(15) $$K^{n}\left(\tau \right)=\exp\left(-in\tau H_{0}^{S}-\frac{n\tau^{2}}{2}\Gamma_{S}+o\left(\tau^{2}\right)\right)=\exp\left[-it\left(H_{0}^{S}-\frac{i\tau }{2}\Gamma_{S}\right)+o\left(\tau^{2}\right)\right]$$

and the resulting non-Hermitian Hamiltonian, effectively acting on *S*, under the Zeno experimental protocol for repeated measurements on the ancilla, may be taken as

(16) $$H_{\mathrm{eff}}=H_{0}^{S}-\frac{i\tau }{2}\Gamma_{S}.$$

At a timescale much greater than τ, the dynamics of the subnormalized density operator $\rho_{S}^{c}\left(t\right)$ is quasi-continuous and, in view of Equation (15), it satisfies the equation

(17) $$\frac{d\rho_{S}^{c}\left(t\right)}{dt}=-i\left(H_{\mathrm{eff}}\rho_{S}^{c}\left(t\right)-\rho_{S}^{c}\left(t\right)H_{\mathrm{eff}}^{†}\right).$$

Equation (12) implies that the quasi-continuous probability p(t) of the successful observation of the desired measurement outcomes (all zeroes) up to time *t* diminishes in time in accordance with the equation

(18) $$\frac{dp(t)}{dt}=\frac{d\;\mathrm{tr}[\rho_{S}^{c}\left(t\right)]}{dt}=-\tau \mathrm{tr}\left[\Gamma_{S}\rho_{S}^{c}\left(t\right)\right],$$

with $\frac{dp(t)}{dt}\leq 0$ because $\Gamma_{S}$ is positive semidefinite.

As commented before, this circumstance is due to the non-Hermiticity of $H_{\mathrm{eff}}$ and the consequent effective non-unitary time evolution operator K(t) for the two-qubit system. It means that we cannot exploit $\rho_{S}^{c}\left(t\right)$ to get relevant statistically valid information about the two-qubit system. In order to have a physically admissible density operator, we simply normalize the reduced, conditional density operator as follows

(19) $$\varrho_{S}^{c}\left(t\right)=\frac{\rho_{S}^{c}\left(t\right)}{\mathrm{tr}[\rho_{S}^{c}\left(t\right)]}=\frac{K^{n}\left(t\right)\rho_{S}\left(0\right){\left(K^{n}\right)}^{†}\left(t\right)}{\mathrm{tr}[K^{n}\left(t\right)\rho_{S}\left(0\right){\left(K^{n}\right)}^{†}\left(t\right)]}=\frac{e^{-iH_{\mathrm{eff}}t}\rho_{S}\left(0\right)e^{+iH_{\mathrm{eff}}^{†}t}}{\mathrm{tr}[e^{-iH_{\mathrm{eff}}t}\rho_{S}\left(0\right)e^{+iH_{\mathrm{eff}}^{†}t}]}$$

which, as we know from Section 2, satisfies the following non-linear evolution equation:

(20) $$\frac{d\varrho_{S}^{c}\left(t\right)}{dt}=-i\left[H_{0}^{S},\varrho_{S}^{c}\left(t\right)\right]-\frac{\tau }{2}\left\{\Gamma_{S},\varrho_{S}^{c}\left(t\right)\right\}+\tau \mathrm{tr}\left[\Gamma_{S}\varrho_{S}^{c}\left(t\right)\right]\varrho_{S}^{c}\left(t\right).$$

It is important to underline that if $\rho_{S}\left(0\right)=|\;\psi_{S}\left(0\right)\;\rangle \langle \;\psi_{S}\left(0\right)\;|$, then the normalized density operator $\varrho_{S}^{c}\left(t\right)$ remains pure during the evolution (has zero entropy) and the corresponding wave function satisfies a non-linear equation

(21) $$i\frac{d|\;\psi_{S}\left(t\right)\;\rangle }{dt}=\left(H_{0}^{S}-\frac{i\tau }{2}\Gamma_{S}\right)|\;\psi_{S}\left(t\right)\;\rangle +\frac{i\tau }{2}\langle \;\psi_{S}\left(t\right)\;|\Gamma_{S}|\;\psi_{S}\left(t\right)\;\rangle \;|\;\psi_{S}\left(t\right)\;\rangle .$$

The protocol leading to the non-Hermitian Hamiltonian given by Equation (16) assumes that the evolution of the combined system S+A is governed by a time-independent Hamiltonian describing the coupling of *S* with the ancilla subsystem *A*. In view of the importance played by the time-dependent Hamiltonian models as control tools, it is worth examining where our protocol fails if the time-independence of *H* is relaxed. This analysis is of course useful to understand the reasons for the restrictions we introduce on *H* and is necessary as well to highlight the possibility of extending this protocol to more general situations.

Let us begin by observing that Equations (9) and (10) are valid in both cases and in particular the formal introduction of the operator K(t). Equations (11) and (13) are, instead, not valid. In fact, if *H* depends on time, in the interval [τ,2τ] the time evolution of (S+A) is ruled out by a Hamiltonian different (due to its time dependence) from that generating the evolution of the system S+A in the interval [0,τ]. This implies that $K^{n}\left(\tau \right)$ must be substituted by the product of *n* generally different operators *K*-like always of argument τ. Moreover, to find the analytical form of *U* may be relatively more complicated than usual.

In practice, the approach required to generate the mathematical expression of $H_{eff}$ is not a trivial extension of the one reported in this paper and turns out to be more intricate. However, the points we have elucidated somehow legitimate the expectation of arriving to a generalized protocol in the near future. In particular, the analytical progress in this problem is achievable in the adiabatic regime, when, in addition to the time-dependent version of the stroboscopic approximation, one assumes that the characteristic frequency of classical fields (controlling Hamiltonian *H*) is much smaller than the measurement repetition rate $\tau^{-1}$.

## 4. Non-Hermitian Hamiltonian Engineering

In Section 3, we considered a scenario in which the experimentalist has a composite quantum system S+A, with some fixed Hamiltonian *H*, on which he repetitively performs projective measurements on qubit *A* only. By exploiting this scheme, we then derived the effective generally non-Hermitian Hamiltonian $H_{\mathrm{eff}}$ describing the quantum evolution of *S*.

In this section, we consider the following inverse problem. Suppose the experimentalist is aimed at implementing the non-Hermitian Hamiltonian $H_{\mathrm{eff}}$ of the system S. Our goal, from a theoretical point of view, is twofold. The first one is to provide the physical Hermitian Hamiltonian *H* to be engineered in the lab for the enlarged system S+A, A being the qubit ancilla A coupled to S. The second one consists of also prescribing the measurement repetition rate $\tau^{-1}$ under which the stroboscopic approximation generates the conditional reduced dynamics of *S*, as governed by the prescribed $H_{\mathrm{eff}}$ of interest. It is worth emphasizing that, to achieve a wider applicability of the method we are going to describe, from the very beginning we assume that $H_{\mathrm{eff}}$ acts on the Hilbert finite-dimensional space of S where it is still representable as a Gauss combination of two Hermitian operators, namely $H_{1}$ + i $H_{2}$. We underline that no assumption is made here concerning the spectra of $H_{1}$ and $H_{2}$ or whether $H_{1}$ + i $H_{2}$ is diagonalizable.

The resolution of the posed inverse problem proceeds as follows. The Hermitian part of $H_{\mathrm{eff}}$ reads $\frac{1}{2}\left(H_{\mathrm{eff}}+H_{\mathrm{eff}}^{†}\right)$ and corresponds to $H_{0}^{S}$ in Equation (14). Calculate the Bohr frequencies for the Hermitian operator $i(H_{\mathrm{eff}}-H_{\mathrm{eff}}^{†})$ and denote by *f* its maximum Bohr frequency. Fix τ in such a way that fτ≪1, e.g., $\tau =10^{-2}f^{-1}$. In contrast to the operator $\Gamma_{S}$ in Equation (14), which is positive semidefinite by construction, the operator $i(H_{\mathrm{eff}}-H_{\mathrm{eff}}^{†})$, as previously claimed, does not possess, in general, such a property. Thus, we introduce the constant c=max(0,−M), where *M* is the minimum eigenvalue of the operator $\frac{i}{\tau }\left(H_{\mathrm{eff}}-H_{\mathrm{eff}}^{†}\right)$. Note that the dimensional parameter *c* depends on the chosen measurement repetition rate τ and that it would vanish if $i(H_{\mathrm{eff}}-H_{\mathrm{eff}}^{†})$. Then, the operator $cI+\frac{i}{\tau }\left(H_{\mathrm{eff}}-H_{\mathrm{eff}}^{†}\right)\geq 0$ and corresponds to $\Gamma_{S}$ in Equation (14). Finally, using the established correspondence and the explicit Formulae (14), we provide the total Hermitian Hamiltonian for the system and the ancillary qubit

(22) $$H=\frac{1}{2}(H_{\mathrm{eff}}+H_{\mathrm{eff}}^{†})⊗|\;0_{A}\;\rangle \langle \;0_{A}\;|+\sqrt{cI+\frac{i}{\tau }\left(H_{\mathrm{eff}}-H_{\mathrm{eff}}^{†}\right)}\;⊗\;\left(|\;0_{A}\;\rangle \langle \;1_{A}\;|+|\;1_{A}\;\rangle \langle \;0_{A}\;|\right).$$

By construction, the maximum Bohr frequency for *H*, which relates the states |0〉 and |1〉 for the ancilla qubit *A*, is of the order $\gamma =\sqrt{f/\tau }$, so it satisfies the condition γτ≪1 if fτ≪1. The latter condition is satisfied as we prescribed the inter-measurement duration time τ accordingly. This validates the stroboscopic approximation.

The proposed scheme for engineering non-Hermitian Hamiltonians at will is rather universal. Whatever finite-dimensional operator $H_{\mathrm{eff}}$ is given (no matter if it is either PT-symmetry or pseudo-Hermitian, no matter if it is diagonalizable or not), there exists a Hermitian operator *H* of twice the dimension (i.e., acting on the tensor product of the original Hilbert space and a qubit ancilla) such that the reduced dynamics of *S* in the stroboscopic approximation are equivalent to the quantum dynamics under the assigned non-Hermitian Hamiltonian $H_{\mathrm{eff}}$. Therefore, Formula (22) explicitly prescribes a Hermitian Hamiltonian for the whole system S+A to simulate, with the help of the experimental protocol described in Section 3, the reduced time evolution of *S*, generated by $H_{eff}$. This last aspect is remarkable since such an inverse protocol is particularly applicable when $H_{eff}$ is pseudo-Hermitian. This means that Equation (22) provides a recipe to generate a Hermitian Hamiltonian and then, generally speaking, a physical scenario where the quantum dynamics of the pseudo-Hermitian Hamiltonian of interest may be simulated. In the class of non-Hermitian Hamiltonian models, pseudo-Hermiticity [52,53,54] occupies a special place since it is the most benign one, with various nice properties as, for example, the existence of invariants or the easy derivation of analytical solutions [55,56].

## 5. Non-Hermitian Dynamics for Coupled Qubits Induced by Repeated Measurements

### 5.1. Symmetric Two-Qubit Effective Non-Hermitian Hamiltonian

**Figure 3:**
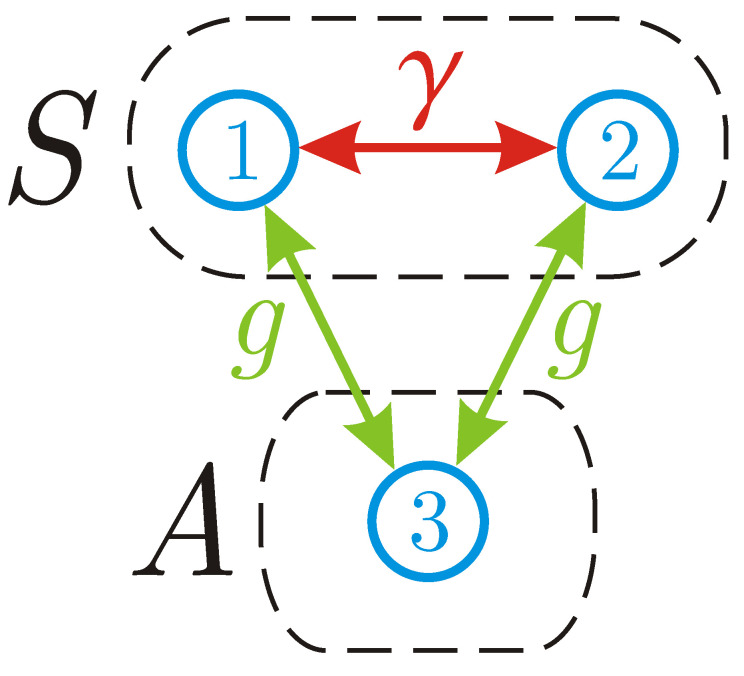
Interaction graph for three spins coupled via Hamiltonian (23). The first two spins compose a bipartite system *S* under study. The third spin is auxiliary (*A*) and is subjected to repeated measurements.

Consider three spin-$\frac{1}{2}$ particles (qubits) with the pairwise interaction Hamiltonian (in units of *ℏ*, that is, ℏ=1)

(23) $$\begin{array}{ccc} H & = & \gamma_{xy}\left(\sigma_{1}^{x}\sigma_{2}^{x}+\sigma_{1}^{y}\sigma_{2}^{y}\right)+\gamma_{z}\sigma_{1}^{z}\sigma_{2}^{z}+g_{xy}\left(\sigma_{1}^{x}\sigma_{3}^{x}+\sigma_{1}^{y}\sigma_{3}^{y}\right)+g_{z}\sigma_{1}^{z}\sigma_{3}^{z}+g_{xy}\left(\sigma_{2}^{x}\sigma_{3}^{x}+\sigma_{2}^{y}\sigma_{3}^{y}\right)+g_{z}\sigma_{2}^{z}\sigma_{3}^{z}, \end{array}$$

which generalizes the so-called XXZ model [57] and assumes that the third auxiliary spin is equidistant from the other two spins; see Figure 3. Hereafter, $\sigma^{x},\sigma^{y}$ and $\sigma^{z}$ denote the conventional set of Pauli operators.

We consider the first two spins as a system (*S*) and the third spin as an ancilla (*A*), whose spin projection onto the *z*-axis is repeatedly measured after equal time intervals τ. If the initial state of the third spin is $|\;0_{A}\;\rangle$, such that $\sigma_{3}^{z}|\;0_{A}\;\rangle =|\;0_{A}\;\rangle$ and the measurements confirm the spin remains in this state, then the system—in the stroboscopic limit—experiences a non-unitary evolution with the effective non-Hermitian Hamiltonian (16)

(24) $$\begin{array}{c} H_{\mathrm{eff}}=\gamma_{xy}\left(\sigma_{1}^{x}\sigma_{2}^{x}+\sigma_{1}^{y}\sigma_{2}^{y}\right)+\gamma_{z}\sigma_{1}^{z}\sigma_{2}^{z}+g_{z}\left(\sigma_{1}^{z}+\sigma_{2}^{z}\right)-i\tau g_{xy}^{2}\left(2I_{12}+\sigma_{1}^{x}\sigma_{2}^{x}+\sigma_{1}^{y}\sigma_{2}^{y}-\sigma_{1}^{z}-\sigma_{2}^{z}\right). \end{array}$$

The contribution of the identity operator $I_{12}$ in $H_{\mathrm{eff}}$ affects only the probability of observing the desired sequence of outcomes, meaning that it does not affect the physical evolution of the normalized density operator $\varrho_{S}\left(t\right)$, cf. Equation (19). Therefore, the physical dynamics of $\varrho_{S}\left(t\right)$ are governed by the non-Hermitian Hamiltonian $H_{\mathrm{eff}}^{\prime }=H_{\mathrm{eff}}+2i\tau g_{xy}^{2}I_{12}$, which reads as follows on a conventional eigenbasis |00〉,|01〉,|10〉,|11〉 of operator $\sigma_{1}^{z}\sigma_{2}^{z}$:

(25) $$\begin{array}{c} H_{\mathrm{eff}}^{\prime }=\left(\begin{array}{cccc} \gamma_{z}+2g_{z}+i2\tau g_{xy}^{2} & 0 & 0 & 0\\ 0 & -\gamma_{z} & 2\gamma_{xy}-i2\tau g_{xy}^{2} & 0\\ 0 & 2\gamma_{xy}-i2\tau g_{xy}^{2} & -\gamma_{z} & 0\\ 0 & 0 & 0 & \gamma_{z}-2g_{z}-i2\tau g_{xy}^{2} \end{array}\right). \end{array}$$

The obtained Hamiltonian provides an adequate description within the stroboscopic approximation, which is justified if $g_{xy}\tau \ll 1$ [48,49,50,51]. However, the quantity $g_{xy}^{2}\tau$ can be comparable with $\gamma_{xy}$ if the coupling strength $g_{xy}\gg \gamma_{xy}$. If this is the case, the anti-Hermitian part of $H_{\mathrm{eff}}^{\prime }$ cannot be neglected and should be properly taken into account.

In a general scenario, the measurement repetition rate $\tau^{-1}$ can be time-dependent, i.e., the duration τ=τ(t) in between the sequential measurements can gradually vary with time *t* on a long timescale (t≫τ(t)). This leads to a time-dependent Hamiltonian $H_{\mathrm{eff}}^{\prime }\left(t\right)$.

### 5.2. Two-Qubit Entanglement Generation

The time evolution operator U(t) of the two-qubit effective time-independent Hamiltonian in Equation(25) can be easily derived. It possesses the same structure as the Hamiltonian and turns out to be precisely

(26) $$U\left(t\right)=\left(\begin{array}{cccc} {}^{-i(\gamma_{z}+2g_{z})t/\hbar }\;e^{2\tau g_{xy}^{2}t/\hbar } & 0 & 0 & 0\\ 0 & \cos\alpha & -i\sin\alpha & 0\\ 0 & -i\sin\alpha & \cos\alpha & 0\\ 0 & 0 & 0 & e^{-i(\gamma_{z}-2g_{z})t/\hbar }\;e^{-2\tau g_{xy}^{2}t/\hbar }, \end{array}\right).$$

with $\alpha =2\left(\gamma_{xy}-i\tau g_{xy}^{2}\right)t\equiv \left(\gamma -ig\right)t$.

Since the dynamics of the two states |00〉 and |11〉 are trivial, we concentrate on the dynamics within the dynamically invariant Hilbert subspace spanned by |01〉 and |10〉 and governed by the 2×2 block. If the two qubits are initially prepared in the pure state $\rho_{S}\left(0\right)=\left|\;01\;\rangle \langle \;01\;\right|$, following the scheme outlined in Section 2 and Section 3, we get

(27) $$\varrho_{S}^{c}\left(t\right)=\frac{\rho_{S}^{c}\left(t\right)}{\mathrm{Tr}\{\rho_{S}^{c}\left(t\right)\}}=\frac{U\left(t\right)\rho_{S}\left(0\right)U^{†}\left(t\right)}{\mathrm{Tr}\{U\left(t\right)\rho_{S}\left(0\right)U^{†}\left(t\right)\}}=\frac{1}{{\left|\cos\left(\alpha \right)\right|}^{2}+{\left|\sin\left(\alpha \right)\right|}^{2}}\left(\begin{array}{cccc} 0 & 0 & 0 & 0\\ 0 & {\left|\cos\alpha \right|}^{2} & i\cos\alpha \sin\alpha^{*} & 0\\ 0 & -i\cos\alpha^{*}\sin\alpha & {\left|\sin\alpha \right|}^{2} & 0\\ 0 & 0 & 0 & 0 \end{array}\right).$$

The (normalized) transition probability towards the state |10〉, $P_{01}^{10}$, is then

(28) $$P_{01}^{10}=\frac{{\left|\sin\alpha \right|}^{2}}{{\left|\cos\left(\alpha \right)\right|}^{2}+{\left|\sin\left(\alpha \right)\right|}^{2}}=\frac{\cos^{2}\left(\gamma t\right)\sinh^{2}\left(gt\right)+\sin^{2}\left(\gamma t\right)\cosh^{2}\left(gt\right)}{\cosh^{2}\left(gt\right)+\sinh^{2}\left(gt\right)}.$$

**Figure 4:**
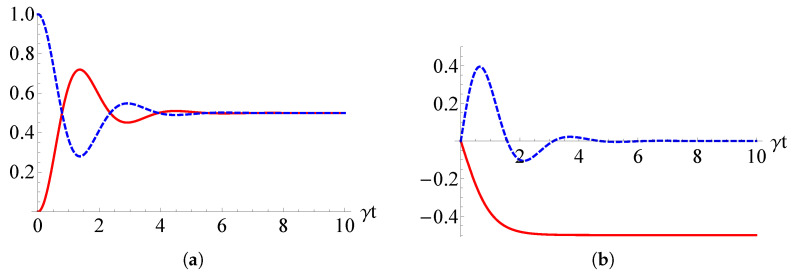
(**a**) Populations of the sates |10〉 (red solid line) and |01〉 (blue dashed line) when the two-qubit system is initially prepared in |01〉 for γ=2g; (**b**) real (solid red line) and imaginary (dashed blue line) parts of the coherence $\langle \;01\;|\varrho_{S}^{c}\left(t\right)|\;10\;\rangle$.

In Figure 4a, the two normalized populations are reported in terms of the dimensionless parameter γt and for γ=2g. The solid red (dashed blue) curve represents the population of the state |10〉 (|01〉) and then the transition probability in Equation (28). We see that both populations reach the value 1/2 at large times. In Figure 4b, instead, we can see the time behaviors of the real (solid red line) and imaginary (dashed blue line) parts of the (normalized) coherence are $\langle \;01\;|\varrho_{S}^{c}\left(t\right)|\;10\;\rangle$. We notice that the coherence does not asymptotically vanish and, rather, it becomes real and equal to −1/2, as it can be easily verified by its analytical expression

(29) $$\langle \;01\;|\varrho_{S}^{c}\left(t\right)|\;10\;\rangle \left(t\right)=-\frac{1}{2}\frac{\sinh(2gt)-i\sin(2\gamma t)}{\cosh^{2}\left(gt\right)+\sinh^{2}\left(gt\right)}.$$

The normalized asymptotic state reached by the two-qubit system turns out to be thus

(30) $$\varrho_{S}^{c}\left(t\to \infty \right)=\frac{1}{2}\left(\begin{array}{cccc} 0 & 0 & 0 & 0\\ 0 & 1 & -1 & 0\\ 0 & -1 & 1 & 0\\ 0 & 0 & 0 & 0 \end{array}\right)=|\;\Psi^{-}\;\rangle \langle \;\Psi^{-}\;|,\;|\;\Psi^{-}\;\rangle =\frac{|\;01\;\rangle -|\;10\;\rangle }{\sqrt{2}},$$

which is one of the the well known maximally entangled Bell states. This result is in accordance with the fact that the generalized von Neumann–Liouville Equation (7) preserves the purity of initial pure states.

In other words, when the system is initially prepared in a pure state, the evolved state, according to Equation (7), remains a pure state [8,10]. Conversely, mixed states change their purity during the time evolution [8,10]. Therefore, we have shown that, by initializing the two qubits in a pure, separable state, we can generate an asymptotic pure entangled state through the procedure described in Section 3 based on repeated measurements on the third ancilla qubit.

### 5.3. Effects of Hamiltonian Anisotropy

The same physical effect of entanglement generation for two qubits, induced by repeated measurements on the third ancilla qubit, does not occur if the system is initialized in either |00〉 or |11〉. This fact is immediately clear from the matrix form of the effective non-Hermitian Hamiltonian (Equation (25)) governing the dynamics of the two coupled qubits. However, it is reasonable to argue that an appropriate generalization of the three-spin model can lead to the appearance of off-diagonal elements ‘connecting’ the two states under consideration. In this way, in the subspace spanned by |00〉 and |11〉 we may have a dynamics similar to the one we brought to light before.

To this end, let us consider the most general model of the three spins, namely

(31) $$\tilde{H}=\gamma_{x}\sigma_{1}^{x}\sigma_{2}^{x}+\gamma_{y}\sigma_{1}^{y}\sigma_{2}^{y}+\gamma_{z}\sigma_{1}^{z}\sigma_{2}^{z}+\alpha_{x}\sigma_{1}^{x}\sigma_{3}^{x}+\alpha_{y}\sigma_{1}^{y}\sigma_{3}^{y}+\alpha_{z}\sigma_{1}^{z}\sigma_{3}^{z}+\beta_{x}\sigma_{2}^{x}\sigma_{3}^{x}+\beta_{y}\sigma_{2}^{y}\sigma_{3}^{y}+\beta_{z}\sigma_{2}^{z}\sigma_{3}^{z}.$$

In this case, the effective non-Hermitian Hamiltonian describing the dynamics of spins 1 and 2, when the repeated-measurement technique is applied on the third spin, turns out to be (up to terms proportional to the identity operator)

(32) $${\tilde{H}}_{\mathrm{eff}}=\left(\alpha_{z}+i\tau \alpha_{x}\alpha_{y}\right)\sigma_{1}^{z}+\left(\beta_{z}+i\tau \beta_{x}\beta_{y}\right)\sigma_{2}^{z}+\left(\gamma_{z}-i\tau \alpha_{z}\beta_{z}\right)\sigma_{1}^{z}\sigma_{2}^{z}+\left(\gamma_{x}-i\tau \alpha_{x}\beta_{x}\right)\sigma_{1}^{x}\sigma_{2}^{x}+\left(\gamma_{y}-i\tau \alpha_{y}\beta_{y}\right)\sigma_{1}^{y}\sigma_{2}^{y}.$$

It is possible to easily verify that this Hamiltonian presents two independent subdynamics: one involving the two states {|00〉,|11〉} and the other involving the two remaining states {|01〉,|10〉}. The existence of these two dynamically invariant subspaces can be traced back to the existence of the following constant of motion $\sigma_{1}^{z}\sigma_{2}^{z}$. In each subspace, thus, the two-spin system effectively behaves like a two-level system and we can write a fictitious two-level Hamiltonian for each subdynamic. The matrix representation of the two-level Hamiltonian ruling the two-spin dynamics within the subspace spanned by {|00〉,|11〉} and {|01〉,|10〉}, respectively:

(33) $${\tilde{H}}_{\mathrm{eff}}^{+}=\left(\begin{array}{cc} \gamma_{z}+\alpha_{z}+\beta_{z}+i\tau \left(\alpha_{x}\alpha_{y}+\beta_{x}\beta_{y}-\alpha_{z}\beta_{z}\right) & \left(\gamma_{x}-\gamma_{y}\right)-i\tau \left(\alpha_{x}\beta_{x}-\alpha_{y}\beta_{y}\right)\\ \left(\gamma_{x}-\gamma_{y}\right)-i\tau \left(\alpha_{x}\beta_{x}-\alpha_{y}\beta_{y}\right) & -[-\gamma_{z}+\alpha_{z}+\beta_{z}+i\tau \left(\alpha_{x}\alpha_{y}+\beta_{x}\beta_{y}+\alpha_{z}\beta_{z}\right)] \end{array}\right),$$

(34) $${\tilde{H}}_{\mathrm{eff}}^{-}=\left(\begin{array}{cc} -\gamma_{z}+\alpha_{z}-\beta_{z}+i\tau \left(\alpha_{x}\alpha_{y}-\beta_{x}\beta_{y}+\alpha_{z}\beta_{z}\right) & \left(\gamma_{x}+\gamma_{y}\right)-i\tau \left(\alpha_{x}\beta_{x}+\alpha_{y}\beta_{y}\right)\\ \left(\gamma_{x}+\gamma_{y}\right)-i\tau \left(\alpha_{x}\beta_{x}+\alpha_{y}\beta_{y}\right) & -[\gamma_{z}+\alpha_{z}-\beta_{z}+i\tau \left(\alpha_{x}\alpha_{y}-\beta_{x}\beta_{y}-\alpha_{z}\beta_{z}\right)] \end{array}\right),$$

where the superscripts + and − refer to the two values ±1 of the constant of motion $\sigma_{1}^{z}\sigma_{2}^{z}$. The operatorial form of ${\tilde{H}}_{\mathrm{eff}}^{\pm }$ in terms of the dynamical variable of a fictitious spin (1/2), omitting terms with no influence on the two-qubit dynamics, reads

(35) $$\begin{array}{cc} {\tilde{H}}_{\mathrm{eff}}^{\pm } & =\Omega_{\pm }\sigma^{z}+\omega_{\pm }\sigma^{x},\\ \Omega_{\pm } & =\alpha_{z}\pm \beta_{z}+i\tau \left(\alpha_{x}\alpha_{y}\pm \beta_{x}\beta_{y}\right)\equiv \mu_{z}+i\nu_{z},\\ \omega_{\pm } & =\left(\gamma_{x}\mp \gamma_{y}\right)-i\tau \left(\alpha_{x}\beta_{x}\mp \alpha_{y}\beta_{y}\right)\equiv \mu_{x}+i\nu_{x}. \end{array}$$

We get the model previously analyzed by putting $\gamma_{x}=\gamma_{y}$, $\alpha_{x}=\alpha_{y}=\beta_{x}=\beta_{y}$, and $\alpha_{z}=\beta_{z}$. We see, in fact, that the first two conditions make the off-diagonal entries in ${\tilde{H}}_{\mathrm{eff}}^{+}$ equal to zero, as expected.

The time evolution operator related to ${\tilde{H}}_{\mathrm{eff}}^{+}$, that is, restricted to the subspace spanned by |00〉 and |11〉, turns out to be

(36) $${\tilde{u}}^{+}\left(t\right)=\left(\begin{array}{cc} \cos\left(\nu t\right)-i\frac{\Omega_{+}}{\nu }\sin\left(\nu t\right) & -i\frac{\omega_{+}}{\nu }\sin\left(\nu t\right)\\ -i\frac{\omega_{+}}{\nu }\sin\left(\nu t\right) & \cos\left(\nu t\right)+i\frac{\Omega_{+}}{\nu }\sin\left(\nu t\right) \end{array}\right),\;\nu =\sqrt{\Omega_{+}^{2}+\omega_{+}^{2}}.$$

We note that, for $\Omega_{+}=0$, we get the analogous form of the time evolution operator in Equation (26).

**Figure 5:**
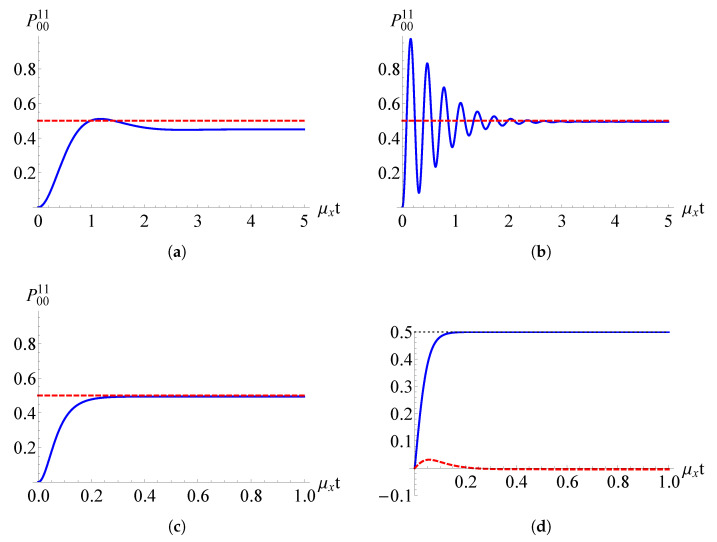
(Color online) (**a**) Populations of the sates |11〉 (solid blue line) when the two-qubit system is initially prepared in |00〉 for (**a**) $\mu_{x}=\nu_{y}=10\mu_{z}=10\nu_{z}$, (**b**) $\mu_{x}=10\nu_{y}=100\mu_{z}=100\nu_{z}$, (**c**) $10\mu_{x}=\nu_{y}=100\mu_{z}=100\nu_{z}$ (the dashed red line represents $P_{00}^{11}=1/2$); (**d**) Real (solid blue line) and imaginary (dashed red line) part of the coherence $\langle \;01\;|{\tilde{\varrho }}_{S}^{c}\left(t\right)|\;10\;\rangle$ when $10\mu_{x}=\nu_{y}=100\mu_{z}=100\nu_{z}$. Plots are reported versus the dimensionless parameter $\mu_{x}\;t$.

In Figure 5a–c, the population of the state |11〉 is reported when the two-spin system is initially prepared in ${\tilde{\rho }}_{S}\left(0\right)=\left|\;00\;\rangle \langle \;00\;\right|$. We can qualitatively appreciate that the different relative weights of the parameters $\mu_{x}$, $\nu_{x}$, $\mu_{z}$, and $\nu_{z}$ give rise to different time behaviors. In all three cases, we chose the favourable condition $\Omega_{+}\ll \omega_{+}$ to generate an asymptotic entangled state. From Figure 5d, in fact, we see that for $10\mu_{x}=\nu_{y}=100\mu_{z}=100\nu_{z}$ (Figure 5c), the coherence of the state ${\tilde{\varrho }}_{S}^{c}\left(t\right)={\tilde{\rho }}_{S}^{c}\left(t\right)/\mathrm{Tr}\left\{{\tilde{\rho }}_{S}^{c}\left(t\right)\right\}$ becomes real at large times, meaning that the two-spin system asymptotically reaches the state

(37) $${\tilde{\varrho }}_{S}^{c}\left(t\to \infty \right)=\frac{1}{2}\left(\begin{array}{cccc} 1 & 0 & 0 & 1\\ 0 & 0 & 0 & 0\\ 0 & 0 & 0 & 0\\ 1 & 0 & 0 & 1 \end{array}\right)=|\;\Phi^{+}\;\rangle \langle \;\Phi^{+}\;|,\;|\;\Phi^{+}\;\rangle =\frac{|\;00\;\rangle +|\;11\;\rangle }{\sqrt{2}}.$$

This result shows that, under the generalized model in Equation (31) and the repeated- measurement procedure, it is possible to generate maximally entangled state in the subspace spanned by |00〉 and |11〉 too. The presence of anisotropy in the exchange interaction between the two spins under consideration and/or between each spin with the ancilla, in fact, makes the half-transition |11〉↔|00〉 possible, thus producing detectable physical effects that would be absent under the more isotropic model in Equation (23). Therefore, it means that, by studying the dynamics in this subspace, we can get information about the level of (an)isotropy of the coupling existing between the two spins and between each spin with the ancilla.

## 6. Conclusive Remarks

Reference [41] reports an original experimental protocol implementing the quantum dynamics of a finite-dimensional system *S* generated by a non-Hermitian Hamiltonian operator. In accordance with this scheme, firstly, *S* is appropriately coupled with a finite-dimensional quantum ancilla subsystem *A* and then the time evolution of *S*, conditioned by a Zeno measurement protocol applied on *A* only, is observed at any intermediate step. In accordance with [41], the reduced density matrix of *S*, stemming from the progression of collapses induced in this way on the state of the combined system S+A, evolves under the action of an effective non-Hermitian Hamiltonian, which may be explicitly constructed in the so called stroboscopic regime limit. In the present work, this method was applied to a system *S* composed by a two-qubit system interacting with a third ancilla qubit. The scope is to demonstrate the effectiveness and usefulness of the protocol to predict the quantum dynamics of the pair of qubits conditioned by a quantum Zeno measurement protocol applied to the ancilla only.

First, we took into account pairwise Heisenberg interactions between the three spins so that the two relevant spins (system *S*) are identically coupled to the ancilla qubit (the case of reflectional symmetry). The method proposed in [41] proved to be successful, leading us to an effective non-Hermitian time-independent two-qubit model. The exact solution of the dynamical problem has been simplified by analyzing the different dynamically invariant subspaces emerging from the symmetry possessed by the effective Hamiltonian. This symmetry-based approach turned out to be useful to study and solve dynamical problems related to more complex interacting spin systems subjected to time-dependent fields [58,59,60,61,62,63,64,65,66]. By focusing our attention on the sub-dynamics involving the two-qubit states |10〉 and |01〉, we brought to light the possibility of generating maximally entangled states of the two qubits. Therefore, we showed that the combined effect of the unitary evolution of (S+A) between two successive conditional measurements on the ancilla qubit can induce quantum correlations on the two-qubit subsystem *S*.

A second interesting aspect consists of the detectable physical effects on the dynamics of the two-qubit system stemming from the isotropy level of the qubit interactions. We know that the type of interaction could considerably affect the system dynamics, giving rise to remarkable physical effects [67,68,69,70]. We generalized the model by analyzing anisotropic Heisenberg interactions between the three qubits. In this case, of course, the effective non-Hermitian two-qubit Hamiltonian turned out to be more complicated. However, conserved symmetries possessed by the Hamiltonian again ensured the existence of two dynamically invariant sub-dynamics, making the study and solution of the two-qubit dynamical problem simpler. We demonstrated that the anisotropic interactions can generate transitions in the subspace involving the two-qubit states |00〉 and |11〉, which were hindered, instead, in the isotropic scenario. So, the possibility of generating maximally entangled states in both sub-dynamics is a transparent and relatively experimentally easy way both to manifest and to get information about the isotropy level of the qubit interactions.

A further important result achieved in this paper is that described in Section 4. It may be described as the inverse of the the protocol adopted in [41]. In fact, starting from a non-Hermitian Hamiltonian model at will for an arbitrary system *S*, it introduces an easy and universal recipe to construct an Hermitian Hamiltonian model for the system S+A where *A* is a qubit system. The importance of this original inverse protocol stems from the fact that it holds whatever the system *S* and its non-Hermitian prescribed model is. When *S* is finite-dimensional, the application of the direct protocol leads to the assigned non-Hermitian Hamiltonian. Thus, for example, we may start from a pseudo-Hermitian Hamiltonian describing a finite system *S*, to generate a physical scenario where the time behavior of *S* may be well simulated under the stroboscopic conditions established in Section 3.

It is interesting to point out that theoretical investigations on non-Hermitian Hamiltonians find useful applications not only in the quantum realm but also in the classical one. Let us think about the non linear optics branch [71], for example. The first optical scenario deserving of a mention is the one regarding the laser-induced continuum structure problem [72], which has been deeply investigated [73] and experimentally confirmed [74]. More recently, instead, a lot of attention has been paid to interacting waveguides. It is possible to show that, under appropriate physical conditions, the dynamics of these systems can be well described by a Schrödinger-like equation where the spatial variable plays the role of time in the standard Schrödinger equation [75]. Notably, experimentalists, through the appropriate choice of materials and laser-based techniques, are able to control some parameters in such a way that the Hamiltonian ruling the dynamics turns out to be non-Hermitian [76,77]. Therefore, in light of these examples, we understand how many intriguing aspects about the dynamics of both quantum and classical physical systems may still need to be found.

A future perspective of the present work could be to investigate the cases in which the parameter τ or the total Hamiltonian for S+A are considered to be time dependent, taking into account the exactly solvable non-Hermitian scenarios recently proposed [15,78]. Moreover, one may wish to concentrate on the application of the theoretical method of [41] in more complex cases, like two-qubit systems immersed in a quantum oscillator environment. In this case, a fruitful comparison with other approaches [79,80,81,82,83] developed to face this kind of problem is possible.
